# Supplementary figures and images for: Fumaric Acids Do Not Directly Influence Gene Expression of Neuroprotective Factors in Highly Purified Rodent Astrocytes
Source: Brain Sci. 2019 Sep 19;9(9):241. doi: 10.3390/brainsci9090241 (PMC6769695; doi:10.3390/brainsci9090241)

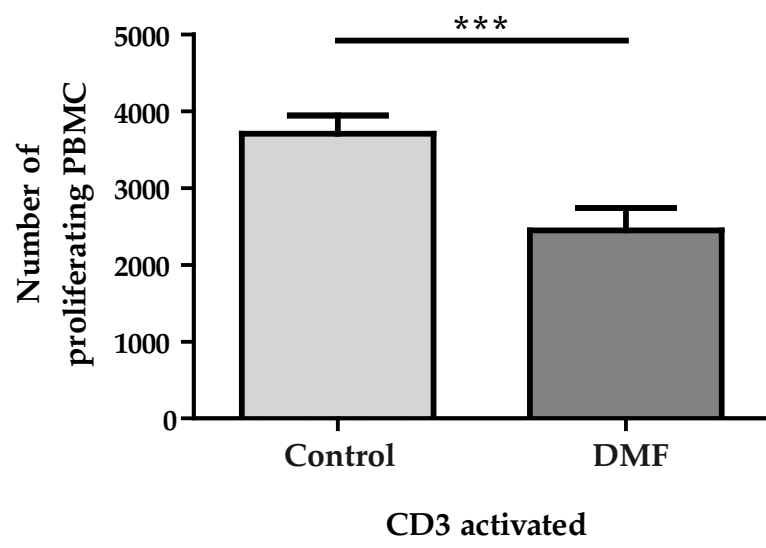

Supplement: Supplementary file 1 [file brainsci-09-00241-s001.pdf]
